# Supplementary material for: Are carbon emissions trading and green financial instruments synergistic? -Comprehensive quantitative research based on content analysis
Source: PLoS One. 2024 Mar 7;19(3):e0298601. doi: 10.1371/journal.pone.0298601 (PMC10919601; doi:10.1371/journal.pone.0298601)
Supplement: S1 Data — (ZIP) [file pone.0298601.s001.zip › Data/地级市Z_F工作报告.zip!/┴╔─■/╕╖╨┬╩╨/└┤╫╘╣1⁄2╓┌║┼í╛┬φ┐╦╩2╛▌═°í┐_2015.docx]

各位代表：

　　现在，我代表阜新市人民政府向大会报告工作，请予审议，并请市政协各位委员和其他列席同志提出意见。

　　一、2014年工作回顾

　　过去一年，我市面临的经济形势严峻复杂，经济下行压力持续加大。在市委的正确领导下，在市人大、市政协的监督支持下，我们紧紧团结全市各族人民，坚持稳中求进工作总基调，全力以赴稳增长、促改革、调结构、惠民生，全市经济社会实现稳步发展，转型振兴迈出坚实步伐。

　　预计地区生产总值626 亿元，增长4.5%；公共财政预算收入71.2 亿元，增长1%；固定资产投资450 亿元，剔除不可比因素增长2.3%；社会消费品零售总额259.4亿元，增长12.5%；实际利用外资2.5 亿美元，增长21.9%；内资到位额310亿元，增长10%；出口总额3亿美元，增长12.5%；城镇和农村常住居民人均可支配收入分别达到21250 元、10527元，分别增长10%。

　　——项目建设取得较大进展。新开工投资五千万元以上项目261 个，其中亿元以上114个；竣工投产248个。10亿元以上项目建设取得突破，总投资36 亿元的鑫河LNG、15 亿元的大金提质改造等项目开工，24亿元的兴隆CBD二期、12亿元的香港豪德商贸城一期等项目竣工，总投资23.4亿元的辉山系列项目部分投产。这些项目为带动投资、稳定增长提供了重要保障。合资合作取得新进展。广厦与宝钢合作、总投资40亿元的建筑产业基地，环宇与中航合作、总投资15 亿元的液压支架项目开工；永生与徐工合作、总投资30亿元的整车装备，睿利与中冶合作的热镀铝生产线项目前期进展顺利。这些项目为提升产业水平、构筑竞争力提供了重要支撑。

　　招商取得新成效。以央企、民企、沪企辽宁行为契机，以京津冀、长三角、珠三角等地区为重点，深化招商合作。分别投资30亿元的天士力产业园、红星国际广场、国际金融港，15亿元的中天钛业、5亿元的东北特钢等92个亿元以上项目签约。这些项目为培育新增长点、促进产业多元发展打下了坚实基础。

　　经贸合作得到加强。成功举办玛瑙博览会、皮革展销会等活动，组织参加广交会、高交会等外贸展会，充分利用韩国周、华商年会、台联台盟等推介活动扩大对外合作。

　　——产业集群建设扎实进展。大唐煤制天然气项目重组稳步推进，瀚石钻机、氟托化工、盈田工业地产一期等项目竣工，聚宝源等3户皮革企业整体搬迁入驻，新能源装机容量新增10万千瓦。国家级高新区、省中小微企业创业基地挂牌，荣获“中国制革示范基地”称号。重点产业集群公共服务平台建设迈出坚实步伐，与辽宁工大、上海有机所、大连化物所、四川大学等合作加深。

　　实施远东铜业等重大工业技改项目40个；新增超亿元企业20户；110 户企业销售收入增长50%以上。新增省级工程技术研究中心4 家、高新技术企业5家。阜矿集团在稳定产量的同时，为全市稳增长做出了积极贡献。

　　——现代农业示范带开局良好。示范带建设完成59.4万亩，在转变农业发展方式、扩大开放、构建科技金融平台、增加农民收入等方面取得突破。伊利饮料、新益达羊毛等项目投产，振隆农产品加工等项目开工。新建、改扩建规模养殖小区63 个。新增节水滴灌77 万亩。农田综合机械化水平达到84%。高标准农田、农村土地整理完成23.5万亩。

　　面对严重旱灾，全市人民同心协力、奋力抗灾自救，加大结构调整、水源调度、人工增雨、劳务输出、保险理赔等力度，努力减少因灾损失、保障受灾群众生活。

　　——服务业加快发展。第三产业增速高于GDP1.5 个百分点，占GDP比重提高1.7个百分点。中央商务区等11个集聚区总营业收入增长20.5%；温泉旅游度假区年接待游客30万人；太平文化旅游集聚区晋升省级行列。京都国际广场、世通物流园一期、浙江大厦等开业运营，紫金财富广场、亚欧国际建材市场等进展顺利。房地产投资130 亿元，销售额64.2亿元。金融机构新增贷款90亿元；盛京银行阜新分行、锦州银行阜新分行、阜新农商银行、阜新银行葫芦岛分行开业；新增2家保险机构、2家证券机构。

　　——城乡环境持续改善。实施十项环境工程。细河综合治理“543”工程全面展开，全流域生态稳步提升；海州露天矿综合治理正式实施；宜居乡村建设扎实推进，164 个行政村环境得到改善；蒙古贞污水处理厂二期开工，温泉新城污水处理厂主体完工；封闭城区自备水源井284眼；实施主城区化工企业、铸造企业转移；完成14项发电企业脱硫脱硝除尘工程；沙化治理25万亩；煤炭物流园铁路项目获批。人工造林66.2万亩，新增城市绿地63 万平方米。国家“双百工程”示范基地入驻企业达到46户。高德矸石山环境综合治理全面展开。阜锦公路改扩建完成，沈彰开发大道通车。改扩建城市道路40 条，完成6条街路雨水收集工程。

　　改造、新建农村公路545 公里。改造城区地下管网103 公里。巴新铁路新邱至大板段铺架完成；京沈客专征地动迁在全省率先完成，阜新段全面建设。

　　启动《市新型城镇化发展总体规划》编制。玉龙、温泉等新城基础设施及重点项目稳步推进。

　　城市环境综合整治年活动效果明显，城市管理更加规范有序，重点街路、小街小巷保洁水平实现提升，66个老旧小区居住环境、物业管理得到改善。

　　——各项改革有序推进。47户工业企业分立生产性服务业，四年任务当年完成。改革工商登记、企业年度验照制度，新增各类市场主体1.4万户。实现服务业水电气热与工业同价。完成公共资源交易607项。

　　全面启动政府转变职能和机构改革。取消、下放行政职权432 项。撤销市级议事协调机构和临时机构466个。新进行政服务中心审批项目14项。公开财政预决算和“三公”经费。完成铁路、邮政、电信业营改增试点工作。整合118条投诉举报热线，开通市12345 政府综合服务热线平台，率先完成省试点任务。

　　推进国家扶贫改革试验区建设，实施新一轮“集团帮村”工程，减少低收入贫困人口6.5万人。彰武县国家土地确权整县推进试点扎实开展，全市土地流转156 万亩。新增农民合作社2570户、家庭农场126 家。林改确权265 万亩。省农村公路改革试点顺利推进。

　　——民生和社会事业全面改善。省、市39件实事全部完成。实施新一轮城镇棚改工程，开工保障性安居工程32611 套，基本建成7360 套。全年发放住房公积金贷款4亿元。

　　实名制就业6.1万人。建立统一的城乡居民基本养老保险制度。启动城镇居民大病保险。新农合筹资标准年人均达到400元。城乡低保、农村五保、重点优抚对象生活补助标准提高11%以上。

　　市中小学生社会实践基地、高专综合楼、卫校实训楼、辽西技师学院实训中心主体完工；海州区义务教育强区通过省验收。万人坑纪念馆维修保护工程全面推进。《查海》舞剧获得省艺术节银奖。电影《谁来陪伴我》在长春电影节获奖。市全科医生临床培训基地、社会养老院改造工程开工，市二院儿科综合楼投入使用。竞走、举重、篮球等项目在省十二运上名列前茅。改扩建134个社区和8个公安派出所办公用房。

　　建设标准化学校32 所，新建、改扩建幼儿园15所，改造维修校舍28.2 万平方米。新建标准化村卫生室134个，改扩建乡镇卫生院38 所。解决2.8万农村人口饮水安全问题。完成503 个行政村亮化工程。建设公益性墓地23座。改造农村危房3786 户。建成100 个农村幸福院。

　　——政府自身建设得到加强。深入开展党的群众路线教育实践活动，269个问题扎实解决。坚持向市委报告工作制度；自觉接受人大法律监督和政协民主监督，214 件建议、209 件提案办复率、满意率100%。

　　深入贯彻落实国务院28 号文件，明确了60项工作任务，逐条逐项推进落实。认真落实了中央巡视组反馈意见专项整改，开展了国务院稳增长、促改革、调结构、惠民生政策落实专项督查。落实了省政府支持企业发展等政策。

　　“六五”普法、法治政府建设深入推进。“五大系统”完成职能转交。第三次全国经济普查登记完成。领导干部接访制度进一步完善。安全生产形势总体良好。平安阜新建设扎实推进。食品药品安全管理稳步提高。市消防培训基地投入使用。民族团结进步事业不断发展，宗教工作和谐稳定。市民兵训练基地一期主体完工，争创全国双拥模范城工作取得新进展。

　　面对经济发展新特征、新规律、新要求，创新工作举措，积极主动作为。围绕稳定增长，制定了支持国家级高新区发展、促进经济增长、促进投资增长、工业企业倍增计划等政策举措；围绕破解难题，实施了“政银保”金融创新、融资渠道拓宽、推行市场化运作、高层次人才平台搭建等创新举措；围绕工作落实，建立了领导包保产业、项目化管理、责任清单、绩效考评过程管理等落实机制；围绕规范管理，健全城市规划审批机制，实行国有建设用地出让价格集体审定制度，明晰农村小型水利设施产权，重新明确学区划分，强化专项审计和领导干部离任审计，促进政府工作效率提高、质量提升、执行力加强。

　　人防、民防、地方志、档案等工作取得新成绩，外事侨务、对台事务、青年事务、妇女儿童、残疾人、红十字会、慈善、老龄等工作开创新局面，国税、地税、电力、通信等工作取得新进展。

　　各位代表，过去一年，在国内经济“三期叠加”、经济运行周期性与结构性因素相互交织的大环境、大背景下，我们能够取得这样的成绩来之不易。这是省委、省政府正确领导、大力支持的结果，是市委统揽全局、科学决策的结果，是市人大、市政协有效监督、鼎力支持的结果，是全市各族人民坚定信心、拼搏进取的结果。在此，我代表市人民政府，向全市各族人民，向人大代表、政协委员，向各民主党派、工商联、无党派人士和各人民团体，向中省直单位、驻阜部队、武警和消防官兵，向所有参与、关心和支持阜新发展的同志们、朋友们，致以崇高的敬意和衷心的感谢！

　　与此同时，我们也认识到经济社会发展中存在的困难和问题。主要是：稳增长基础薄弱，产业结构性矛盾突出，传统产业持续低迷，接续替代产业支撑不足，新的增长点不够；统筹促进发展、改善生态、保障民生压力加大，项目引进难度增大，有效投资进度放缓，企业生产经营面临困难较多，政府债务进入偿还高峰期，资源环境约束明显加大，解决历史遗留问题等民生任务繁重；农业抵御自然灾害能力较弱，服务业业态发展不均衡；政府系统个别部门服务意识薄弱、办事效率较低、创新能力不足、工作质量不高。对于这些问题，我们一定清醒认识、积极应对、认真解决。

　　二、2015年工作安排

　　当前，我国经济发展进入新常态，主动适应新常态、开创转型振兴新局面，是我市当前乃至长期必须遵循的重要指导。适应新常态，要把握发展新内涵，既关注数量上增与减、速度上快与慢的统筹协调，更注重稳增长和调结构的平衡、质量和效益的同步提升、激活存量和优化增量的并举发力，在稳增长中讲质量、求效益，在促发展中转方式、调结构。适应新常态，要认识发展新特征，打破惯性思维，转变工作理念，创新工作举措，主动顺应新形势，积极体现新作为，努力开创新局面。适应新常态，要抢抓发展新机遇，把握东北全面振兴、“一带一路”、京津冀协同发展的战略机遇和央企、民企、沪企辽宁行的合作契机，挖掘新潜力，增强新动力，培育新增长点。

　　适应新常态，要贯彻发展新要求，推进国务院28号文件和稳增长、促改革、调结构、惠民生，以及省委、省政府新一轮突破辽西北政策措施落实，立足大局，顺势而为，积极作为，坚定不移地推动全市经济社会发展提质增效、转型升级。

　　今年政府工作的总体要求是：深入贯彻落实党的十八届三中、四中全会和中央、省经济工作会议精神，按照市委十一届七次全会部署，积极主动适应新常态，紧紧围绕转型振兴总目标，坚持稳中求进工作总基调，以改革创新为动力，以全民创业、万众创新为保障，加快构建现代产业体系，全力抓好工业产业集群、现代农业示范带、服务业集聚区和城镇化建设，着力保障和改善民生，扎实推进法治政府建设，努力开创转型振兴新局面。

　　主要预期目标是：地区生产总值675 亿元，增长6.1%；公共财政预算收入72.7 亿元，增长2.1%；固定资产投资477 亿元，增长6%；社会消费品零售总额288亿元，增长11%；城镇和农村常住居民人均可支配收入分别达到23160元、11470 元，分别增长9%。新的一年,我们要突出经济发展、改革创新、生态建设、惠及民生工作主题，更加注重稳定增长，以提高经济发展质量和效益为中心，保持增长合理区间，统筹经济效益、民生效益、生态效益协调发展；更加注重改革创新，坚持改革驱动、市场驱动、创新驱动、开放驱动，不断增强发展的内生动力；更加注重结构调整，扩大增量调结构、激活存量稳增长，大力发展实体经济，在传统产业转型升级、接续替代产业壮大、新兴产业培育、现代服务业发展、新型城镇化建设、农业发展方式转变上培育新增长点；更加注重改善民生，量力而行，尽力而为，兜底线补短板，着力解民忧、惠民生、促和谐；更加注重工作落实，坚持实事求是、埋头苦干、攻坚克难，扎扎实实推进各项工作高质高效完成。

　　（一）坚持以项目建设为核心，培育新的增长点

　　继续开展“项目建设年”活动。新开工投资五千万元以上项目200 个，其中亿元以上100个；竣工投产200个。突出抓好三次产业重点项目建设，尽快形成大项目支撑、带动作用。在工业项目上，积极推进煤制天然气一期、远东铜业、大金提质改造、康博太阳能电池等项目竣工投产，加快宝钢建筑产业基地、鑫河LNG、国能煤焦油、中国航天液压、力达铸造二期等项目进程，开工建设东北特钢、凯莱英医药、天士力产业园等项目；以中天钛业项目为龙头发展钛产业；推动中国轻工业进出口总公司制革加工、中电集团变压器生产基地、中国清洁能源公司LNG 等项目落地。在服务业项目上，推进紫金财富广场一期、香港豪德商贸城一期、亚欧国际建材市场、宝地·太阳广场等项目投入运营，加快兴隆CBD三期等项目建设，红星国际广场等项目取得新进展。在农产品加工项目上，推进振隆农产品加工等项目竣工投产，加快建设辉山奶粉、雨润畜禽产品产业园等项目。

　　深化合资合作。加快推进永生与徐工、环宇与中航等开展全面合作，支持企业在品牌引进、战略重组、海外并购、产业协作配套上实现突破。

　　提高项目工作水平。继续实行重大项目包保机制，及时解决问题，加快项目建设进度，确保尽快竣工、尽快转为企业。把握东北振兴政策落实、国家产业政策导向，包装储备一批重大项目。完善产业园区环境保护体系，严格环保准入。

　　（二）坚持以工业经济为支撑，推进产业扩规模、增效益

　　促进产业集群提升。全力推进煤化工产业集群发展，在加快现有项目建设的同时，着力引进建设一批主副产品配套项目。坚持液压产业集群整机化、高端化、品牌化发展，形成集聚效应，提升知名度。打造氟化工产业集群特色精细化工品牌，扩大产业影响力。提升皮革产业集群加工能力，建设劳动力转移就业集聚区。提高板材家居产业集群产品附加值，进一步发挥省中小微企业创业基地作用。推进国家新能源示范市创建工作。加快各类产业园区发展，实现水电气暖、污水处理等基础设施更加完善，研发检测、金融担保、交易网络等平台基本建成。

　　支持企业做强做大。新增超亿元企业20户；销售收入增长50%以上企业100户；规模以上工业增加值增长5.5%。鼓励和支持企业科技创新，提高税收贡献率。

　　深化市矿合作，全力支持阜矿集团稳定产量、增加效益、多元发展，发挥对全市经济的骨干作用。

　　推进传统产业转型升级。加大企业技术改造力度，支持企业开发新技术、新工艺、新产品，提高产品附加值，形成核心竞争力。改造升级装备制造、能源、建材等产业，促进产品创新、管理创新、商业模式创新。

　　积极培育新兴产业。以承接产业转移、龙头企业牵动为重点，引进培育一批新兴产业。推进国家级高新区建设，在产业升级、企业壮大、技术创新、品牌打造上实现突破。做好国家首批“两化”融合试点工作。

　　全力抓好经济运行。继续实行领导干部联系企业制度，坚持问题导向、因企施策、一企一策，保障企业正常生产经营。开展政策、融资、人才、产品等对接活动，支持企业拓宽市场、扩大销售，研发适销对路新产品。

　　（三）坚持以示范带建设为载体，加快发展现代农业

　　扎实推进示范带建设。突出果品经济林、精品农业、高效农业、设施农业发展，新建示范带63.5 万亩。积极发展多种形式的适度规模经营，支持新型农村合作组织和致富带头人发展；加大农业开放力度，引进项目100个以上；推进水电路等基础设施建设；着力培育特色品牌，积极打造无公害、绿色、有机产品；加快科技信息、冷链物流、金融平台、专业市场等服务体系建设。

　　稳步发展传统农业。加快推进种植业结构调整，优化农作物布局，提高农产品质量和效益。推进农业产业化经营，培育壮大一批龙头企业。稳定粮食和主要农产品产量，加强农业技术推广，增强避灾、抗灾能力。

　　继续推进畜牧业强市战略。新建、改扩建规模养殖小区50个，推动畜牧业向规模化、标准化、产业化方向发展。

　　增强农业综合生产能力。完成节水滴灌9 万亩。实施高标准农田、农村土地整理46万亩。实现农业保险面积170万亩。推广农机具1万台套。做好人工影响天气工作。

　　抓好扶贫工作。以现代农业示范带、“集团帮村”和驻村工作队为载体，创新“政银保”金融扶贫、互助式扶贫机制，实施精准扶贫，抓好国家扶贫改革试验区工作，减少低收入贫困人口5万人。

　　（四）坚持以集聚区建设为抓手，促进服务业提质升级

　　提高集聚区发展水平。培育壮大中央商务区、温泉旅游度假区等5 个省级服务业集聚区，充分发挥促消费、扩内需作用。促进各类特色服务业集聚区尽快建成。加快专业市场建设步伐。

　　促进现代服务业发展。推动现代物流、电子商务、信息科技等服务业加快发展。加快世通二期、皮革等物流园建设，开工建设煤炭物流园、大玉冷链物流等项目。

　　提升金融业发展水平。新增贷款70亿元；积极引进股份制金融机构及保险公司、证券公司；推进一批金融集聚项目；力争振隆、德尔上市；规范小额贷款公司管理。实施金融帮扶小微企业工程。创造良好的金融生态环境。

　　促进旅游业发展。做好工业遗产旅游开发，完善海棠山、瑞应寺旅游区建设。推进宝地温泉小镇等项目。扩大“中国玛瑙之都”、敖包文化等特色旅游品牌影响力。促进乡村旅游休闲消费。

　　（五）坚持以生态建设为保障，着力打造宜居环境

　　1、细河综合治理工程。推动“543”工程完工，实现细河断面水质稳定达标；重点做好绿化、湿地等生态建设，将细河沿岸打造成生态带；改造维护城市段，规划桥梁建设，完善基础设施、绿化美化等配套工程，营造良好休闲健身活动空间；启动全流域生态综合治理工程，着力打造生态怡人、环境优美、人水和谐的母亲河。

　　2、海州露天矿综合治理工程。启动扬尘治理、生态绿化建设，实现残煤自燃、滑坡危害有效控制。通过今后几年的不懈努力，实施污染治理、生态建设、遗产保护三大工程，着力打造绿色生态、环境宜居的城市品牌。

　　3、空气环境治理工程。完成“蓝天工程”等大气污染减排工作。推进国家“双百工程”示范基地建设。完成热电企业脱硫脱硝除尘工程。推进建成区集中高效供暖，逐步取缔分散供暖锅炉。积极开展主城区粉煤灰、煤矸石、露天储煤场、尾矿等综合清理整治专项行动，实现扬尘有效治理、空气质量不断改善。

　　4、推进污水处理厂建设运行。蒙古贞二期、氟产业开发区、温泉新城、津源二期、清源中水回用等投入使用，启动太平区等污水处理厂建设，完善经营保障机制，实现县区、重点产业基地污水处理设施全面建成、稳定运行。划定集中式水源保护区，实施保护区内污染源清理。加强城区自备水源井关闭后续管

理。

　　5、做好宜居乡村建设。以村容整洁为目标，以垃圾治理为重点，以健全管护长效机制为保障，实现全部行政村环境卫生整治达标，逐步形成一批宜居示范村。

　　6、抓好绿化沙化治理。完成人工造林12.4万亩、封山育林5万亩；沙化治理24 万亩；城区植树12 万株，新增绿地20万平方米；闭坑矿山治理483亩，矿区绿化1000亩。

　　（六）坚持以基础设施建设为重点，提高城乡建设管理水平

　　加强基础设施建设。将地下管网改造作为一项重要民生工程、保障工程，开展供热管网改造攻坚年活动，改造供热管网80公里，提高城市供热主管网保障能力；改造老旧住宅小区供水二次加压泵房10 座；推进城市燃气危旧管网改造；巩固老旧小区管网改造成果。实施城市道路改扩建、街路整治、雨水收集工程。辽西危废处置中心投入使用。启动市垃圾处理厂三期工程，新建污泥处理池。开工建设500千伏输变电工程。推进“数字阜新”建设。巴新铁路通车，启动通辽连接京沈客专彰武段快速铁路前期工作，配合京沈客专、辽西北供水、锦阜高铁路建设。

　　积极稳妥推进城镇化。加快玉龙新城在建项目进度，促进一批商住项目尽快竣工、一批公共服务项目收尾并实施市场化运营；重点抓好教育、医疗、社会服务等公共设施建设，启动实验小学玉龙分校和幼儿园建设，推进博乐教育基地项目，优化玉龙新城学区划分，完善和提升市第三人民医院；加快京沈客专阜新站周边开发。温泉新城实现道路、公交和绿化、亮化等配套设施全面完善，初具规模形象。以完善城市发展布局、集聚效应提升为重点，做好老城改造。抓好县城和中心镇、重点镇建设，增强集聚能力。促进房地产业平稳健康发展。以去库存、调结构、补配套为重点，加快续建项目建设，尽快转为功能完备小区，调动激活房地产市场，消化存量住房。

　　强化城市管理。牢固树立“三分建设、七分管理”的理念，防止“重建设、重投入、轻管理”现象，继续完善城市网格化、属地化管理体制，建立健全门前三包、卫生保洁、交通秩序、设施维护等管理制度，做到监管无盲区、责任无死角，并形成长效机制。坚持规划先行、市场化运作、项目化管理的原则，提高绿化、亮化、硬化标准和水平。加大城市环境综合整治力度，着力打造干净整洁、绿色宜居、规范有序的城市环境。

　　科学编制规划。坚持分类指导、因地制宜、产城互动的原则，完成《市新型城镇化发展总体规划》、《土地利用总体规划》编制和《城市总体规划》修编。

　　（七）坚持以改革创新为引领，增强发展活力

　　加大简政放权力度。继续抓好上级行政职权下放承接工作，做到应放尽放。加强市级保留审批事项的后续监管、优化流程、提质提效。加大对下放县区审批事项的培训和指导，做到下放和承接运转顺畅。

　　扎实推进政府机构改革，进一步理顺权责关系，规范机构设置。重新制定市政府部门“三定”规定。推进事业单位改革和法人登记制度改革。进一步规范管理现有议事协调机构和临时机构。做好机构编制人员实名制动态管理。抓好各级机关所属企业脱钩改制工作。组织推进“三证合一”。

　　深化财税体制改革。贯彻落实新《预算法》，清理规范行政事业性收费，严控财政新增支出和“三公”经费支出。严格执行市本级政府债务偿债准备金制度，全面落实“营改增”工作。巩固拓展现有税源，培育发展新兴税源，提高财政收入质量和能力。推进机关事业单位养老保险制度改革。启动实施公车改革。积极稳妥做好农村各项改革。

　　推进农村土地确权登记颁证工作，做好市县乡村四级土地流转平台建设，促进土地经营权公平、有序流转。发展市级专业合作示范社50 家。抓实民生和社会事业改革。推进县级公立医院综合改革。继续实施基本药物制度。做好公交运营改革和农村公路监督体制改革。

　　加快发展民营经济。扶持壮大中小微企业，推进民间资本进入实体经济和基础设施、公用事业、社会事业等领域。

　　不断强化工作创新。把创新体现在政策落实、决策执行、机遇把握上；体现在解决问题、破解难题、攻坚克难上；体现在产业升级、项目建设、企业运行、要素保障、生态建设、民生改善上；体现在效率提升、质量提高、服务优化上；体现在社会公认、群众满意上，发挥创新引领作用。

　　（八）坚持以开放为驱动，全面推进对外合作

　　锲而不舍抓招商促合作。紧紧抓住“一带一路”、京津冀协同发展战略和京沈客专建设等机遇，以央企、民企、沪企辽宁行为契机，加强经贸合作、项目对接、产业配套、企业融合，打造产业转移承接地。加大面向国内外500强和行业龙头企业的招商力度，积极引进高端产业、优质项目、核心技术和总部经济。实际利用外资5700 万美元（新口径），增长7.5%；内资到位338亿元，增长10%。

　　创新招商合作机制。主动适应新常态下企业投资需求，以市场为导向促进对接合作。发挥企业招商主体作用,大力开展以商招商、专业招商、协会招商。推进招商制度化、常态化，深入开展驻地招商、自主招商、精准招商。规范清理现有招商政策。积极搭建“走出去”、“请进来”平台，优化投资环境，提高服务水平。

　　加强区域合作。深入参与“一带一路”、京津冀协同发展，主动融入沈阳经济区合作发展，全面推动与蒙东地区一体化发展。

　　促进外经外贸合作，搭建企业产品、技术合作平台。培育省级出口基地，丰富出口产品，扩大出口规模。出口总额3.1亿美元，增长5%。

　　（九）坚持以改善民生为根本，统筹社会事业发展

　　全力办好市25件民生实事（附后），确保当年实施、当年完成、当年见效受益。全面落实省政府确定涉及阜新的实事工程。

　　建成保障性安居工程2.4万套以上，新开工棚改工程1000套。改造农村危房2600户。

　　着力培养和引进专业型、适用型、技能型人才，发挥好高层次人才平台及政府顾问作用。深化市校合作，促进辽宁工大科技和人才向重点产业集聚。

　　千方百计保持就业稳定。做好高校毕业生、困难群体就业工作，全年新增城镇实名制就业4万人。完善全民社会保障体系。进一步扩大社会保险覆盖面。健全城镇居民、农村居民大病保险制度。提升各级各类教育功能。提高教育教学质量，促进教育公平均衡发展。市中小学生社会实践基地投入使用。

　　完善公共文化服务体系。积极开展群众文化活动，加快文化产业发展。完成万人坑纪念馆维修保护工程建设，发挥爱国主义教育基地功能。推进城区数字电视整体转换、社区书屋建设工作。加强基本公共卫生服务建设。

　　提高全市卫生技术水平和服务质量。市全科医生临床培训基地投入使用，市中心血站、卫生监督所主体完工。加强农村医疗卫生服务体系建设。新农合筹资标准年人均达到460元。继续做好农村改厕工作。完善公共体育服务设施网络，促进全民健身活动蓬勃开展。做好篮球品牌赛事活动，提升“篮球名城”品牌影响。

　　努力构建和谐劳动关系。完善妇女创业就业服务体系。做好大学生志愿服务工作。促进各级慈善事业健康发展。残疾人康复中心投入使用。推进国家进村入户信息服务工程。建设“农超对接”社区便民连锁店10 家、“新网工程”示范店10 家。

　　继续推进平安阜新建设，完善社会治安防控体系。深化矛盾纠纷排查，强化信访积案化解力度。全面贯彻落实新《安全生产法》，坚决杜绝重特大事故发生。做好食品安全工作支持民族地区快速发展。依法管理民族宗教事务，增进民族团结。努力争创全国双拥模范城。做好第九届社区换届选举工作。科学编制“十三五”规划。

　　（十）坚持以依法行政为准则，加强政府自身建设

　　严格依法行政。全面贯彻落实国家、省和市委各项决策部署，确保政令畅通、令行禁止。坚持定期向市委报告工作制度。自觉接受人大法律监督、询问和政协民主监督、评议，强化审计、监察、司法等专门监督，主动接受社会监督、舆论监督。认真办理好人大代表建议和政协委员提案，切实提高办理质量和满意率。深入落实依法治市要求，坚持法不授权不可为、法定职责必须为，坚决纠正不作为、乱作为，坚决克服懒政、怠政，坚决惩处失职、渎职。完善依法决策机制，推进决策集体审议制度。坚决做到“讲诚信、懂规矩、守纪律”，政府作出的承诺，必须一一兑现、逐项交账。深化政务公开，建立政府权力清单、负面清单、责任清单制度。

　　促进提质提效。坚持把改革创新作为适应新常态、引领新常态的硬手段、硬措施，转变工作理念，创新思维方式，推动政府工作取得新成效。坚持高标准、高质量推进工作，做到过程与结果、效率与质量、成本与效益相统一。倡导雷厉风行、一抓到底的工作作风，加强工作项目化、精细化管理，以时限倒逼进度，以目标倒逼责任，有序限时推进落实。优化行政服务中心功能，抓好网上审批平台建设，不断提高政府部门工作效率、办事质量、服务水平。完善政府绩效管理体系，促进动态考核、过程管理、结果评估科学。

　　坚持务实为民。保持尽职尽责、勇于担当的工作态度，在经济运行、产业发展、项目建设上锲而不舍，在生态建设、民生改善、社会和谐上尽心尽力，在问题解决、工作落实上善于攻坚。坚持实事求是、真抓实干的工作作风，抓工作、谋发展要算好良心账、民心账、效益账，取得实实在在的工作成效。树立正确政绩观，既立足当前、干好当年，更筑牢实体经济、生态建设等长远基础。巩固和拓展教育实践活动成果，带着感情和责任做好群众工作，真正打通服务群众“最后一公里”。充分发挥市12345政府综合服务热线平台作用，进一步畅通民意表达渠道，确保群众合理诉求得到有效解决。

　　恪守廉洁奉公。继续严格执行中央八项规定和省委、市委十项规定，自觉履行“三严三实”要求，推进作风建设规范化、常态化、长效化。厉行勤俭节约，反对铺张浪费。加强公共资源交易监管，营造公平公正的环境。开展行业纠风专项治理。进一步强化领导干部经济责任审计监督，严肃查处违法违纪案件。加强公务员队伍建设，努力打造克己奉公、勤勉尽责、人民满意的公务员队伍，营造干部清正、政府清廉、政治清明的良好局面。

　　各位代表，加快转型振兴责任重大、使命光荣。让我们在市委的领导下，团结和带领全市各族人民，坚定信心，奋发有为，适应新常态，推动新发展，为实现转型振兴宏伟目标而努力奋斗！
